# Role of cystine transport in intracellular glutathione level and cisplatin resistance in human ovarian cancer cell lines

**DOI:** 10.1038/sj.bjc.6600786

**Published:** 2003-03-18

**Authors:** S Okuno, H Sato, K Kuriyama-Matsumura, M Tamba, H Wang, S Sohda, H Hamada, H Yoshikawa, T Kondo, S Bannai

**Affiliations:** 1 Department of Obstetrics and Gynecology, Institute of Clinical Medicine, University of Tsukuba, Tsukuba, Ibaraki 305-8575, Japan; 2 Department of Biochemistry, Institute of Basic Medical Sciences, University of Tsukuba, Tsukuba, Ibaraki 305-8575, Japan; 3 Department of Biochemistry and Molecular Biology in Disease, Atomic Disease Institute, Nagasaki University School of Medicine, Nagasaki 852-8523, Japan

**Keywords:** cystine, transporter, glutathione, drug resistance, cisplatin

## Abstract

Transport system x_c_^−^ is a member of plasma membrane heterodimeric amino-acid transporters and consists of two protein components, xCT and 4F2hc. This system mediates cystine entry coupled with the exodus of intracellular glutamate and regulates the intracellular glutathione (GSH) levels in most mammalian cultured cells. We studied the activity of system x_c_^−^ and GSH content in human ovarian cancer cell line (A2780) and its cisplatin (CDDP)-resistant variant (A2780DDP). The rate of cystine uptake was approximately 4.5-fold higher in A2780DDP cells than in A2780 cells and the cystine uptake in A2780DDP cells was mediated by system x_c_^−^. Intracellular GSH content was much higher in A2780DDP cells but it fell drastically in the presence of excess glutamate, which inhibited the cystine uptake competitively. xCT and 4F2hc mRNAs were definitely expressed in A2780DDP cells, but far less in A2780 cells. Expression of system x_c_^−^ activity by transfection with cDNAs for xCT and 4F2hc made A2780 cells more resistant to CDDP. Similar results on the cystine uptake were obtained in human colonic cancer cell lines. These findings suggest that the system x_c_^−^ plays an important role in maintaining the higher levels of GSH and consequently in CDDP resistance in cancer cell lines.

Cisplatin (cis-diaminedichloroplatinum (II), CDDP) is one of the most widely used chemotherapeutic agents for the treatment of human ovarian cancer and other tumours (Rosenberg, 1985). CDDP is able to exert its cytotoxity to ovarian cancer via the formation of intra- and interstrand CDDP-DNA adducts, which can ultimately result in cell cycle arrest at G1, S, or G2-M and the induction of genetically programmed cell death (Reed, 1998). One of the major problems with CDDP treatment is the clinical development of resistance to this drug (Perez *et al*, 1993). Although the mechanisms underlying tumour resistance to CDDP *in vivo* have not been understood clearly, *in vitro* studies on cell lines have shown that they are multifactorial. These include decreased drug transport, increased cellular detoxification because of increased glutathione (GSH), changes in DNA repair involving increased nucleotide excision repair and/or loss of mismatch repair, increased tolerance of DNA adducts, and alterations in the apoptotic cell death pathway (Andrews and Howell, 1990; Goto *et al*, 1995; Johnson *et al*, 1997; Li *et al*, 1999, 2001). However, there is abundant evidence showing that increase in GSH is a major factor in CDDP resistance and several CDDP-resistant cell lines keep the higher levels of GSH than the sensitive cell lines (Behrens *et al*, 1987; Mistry *et al*, 1991; Godwin *et al*, 1992).

The biosynthesis of GSH occurs in two ATP-dependent reactions (Meister, 1988). The first step is the synthesis of *γ*-glutamylcysteine (*γ*-GC) from cysteine and glutamate catalysed by *γ*-glutamylcysteine synthetase (*γ*-GCS). The second step is the synthesis of GSH from *γ*-GC and glycine catalysed by GSH synthetase. *γ*-GCS is the rate-limiting enzyme and the increase in its activity results in the increase in intracellular GSH. It has been shown that the activity of *γ*-GCS is higher in CDDP-resistant cells than in CDDP-sensitive cells and this may contribute to the high GSH levels in the former cells (Goto *et al*, 1995; Yao *et al*, 1995; Iida *et al*, 1999). Cysteine is an essential amino acid for many types of mammalian cultured cells, but it is unstable in the extracellular fluid, for example, in culture media and is rapidly autoxidised to cystine (Toohey, 1975). Plasma membrane transport of amino acids is mediated by several transport systems (Christensen, 1990). We described previously in cultured mammalian cells an Na^+^-independent anionic amino-acid transport system, designated system x_c_^−^, highly specific for cystine and glutamate (Bannai and Kitamura, 1980, 1981; Bannai, 1986). System x_c_^−^ is a heterodimeric amino acid transporter consisting of two protein components named xCT and 4F2hc (Sato *et al*, 1999, 2000). Cystine taken up via system x_c_^−^ is rapidly reduced to cysteine, which is used for the synthesis of GSH (Bannai and Ishii, 1982). Since intracellular cysteine is a rate-limiting precursor for GSH synthesis, the intracellular GSH level is regulated by the system x_c_^−^ activity. In the present work, we have investigated the cystine transport activity in the CDDP-resistant and CDDP–sensitive human ovarian cancer cell lines.

## MATERIALS AND METHODS

### Cell culture

Human ovarian cancer cell line (A2780 and its CDDP-resistant variant A2780DDP) and human colonic cancer cell line (HCT8 and its CDDP-resistant variant HCT8DDP) were donated by Dr K J Scanlon (Biochemical Pharmacology, City of Hope National Medical Center, Duarte, CA, USA). They were cultured in RPMI 1640 supplemented with 5% fetal bovine serum at 37°C in 5% CO_2_. The CDDP-resistant cells were treated with 100 *μ*M CDDP (Nihon Kayaku Co., Tokyo, Japan) for 2 h every week as described (Iida *et al*, 1999).

### Uptake of cystine

The uptake of cystine was measured by techniques described previously (Bannai and Kitamura, 1980). Cells were plated at 5 × 10^5^ in a 35 mm diameter dish and cultured for 24 h. They were rinsed three times in warmed phosphate-buffered saline (pH 7.4) containing 0.9 mM CaCl_2_, 0.5 mM MgCl_2_ and 5.6 mM glucose (PBSG). The cells were then incubated in 0.5 ml of the prewarmed uptake medium for 2 min at 37°C. The uptake medium was PBSG containing [^14^C]cystine (NEN Life Science Products Inc., Boston, USA) (0.1 *μ*Ci in 0.5 ml). When Na^+^ dependency of the uptake was examined, the uptake of cystine was measured in an Na^+^-free medium, in which Na^+^ was replaced by choline. The uptake was terminated by rapidly rinsing the dish with ice-cold phosphate-buffered saline and the radioactivity in the cells was counted. The rate of uptake was determined under conditions approaching the initial uptake rate, that is, by taking the values for the 2 min uptake of cystine. A portion of the cell lysate was used for determining the radioactivity and another portion was assayed for protein.

### Cytotoxicity of CDDP

The sensitivity of cells to CDDP was determined by dye exclusion method using nigrosin (Patterson, 1979).

### Determination of intracellular glutathione

The glutathione content was measured by enzymatic method described previously (Bannai and Ishii, 1982), which is based on the catalytic action of glutathione in the reduction of 5,5′-dithiobis(2-nitrobenzoic acid) by the glutathione reductase system (Tietze, 1969). Cells were plated at 5 × 10^5^ in a 35 mm diameter dish and cultured for 24 h and total glutathione (GSH and GSSG) was extracted with 5% trichloroacetic acid. The glutathione extracted from the cells was mostly GSH and the content of the oxidised form, GSSG, was negligibly low throughout this study.

### Determination of intracellular cysteine

The cysteine content in the cells was determined by the method of Cotgreave and Moldeus (1986) with slight modifications. Briefly, the cells, plated at 5 × 10^5^ in a 35 mm diameter dish and cultured for 24 h, were rinsed three times with PBSG and incubated in the dark at room temperature for 10 min with 100 *μ*l of 8 mM monobromobiamine in 50 mM *N*-ethylmorpholine, pH 8, and 100 *μ*l of PBSG. Then 10 *μ*l of 100% trichloroacetic acid was added. The protein precipitate was removed by centrifugation at 3000 **g** for 5 min and aliquots were analysed for cysteine-bimane adducts by HPLC. The HPLC separation was achieved on a steel column (4.6 mm × 100 mm) packed with 3 *μ*m octadodecylsilica reversed-phase material purchased from Nacalai Tesque Inc., Kyoto, Japan. The fluorescence at 480 nm was monitored with the excitation at 394 nm. The elution was performed with 9.0% (v/v) acetonitrile in 0.25% (v/v) acetic acid, pH 3.7, for 8 min, and then with 75% (v/v) acetonitrile in water for 5 min. The flow rate was 1 ml min^−1^ throughout the process.

### Northern blot analysis

Total cellular RNA was extracted using ISOGEN purchased from Nippon Gene, Toyama, Japan. The cDNA probes for human xCT and 4F2hc were labeled with [*α*-^32^P]dCTP using Rediprime™II random prime labelling system from Amersham Pharmacia Biotech (Little Chalfont, UK). Total RNA was electrophoresed on a 1% agarose gel in the presence of 2.2 M formaldehyde, transferred and hybridised as described previously (Sato *et al*, 1999).

### Cell transfections

The inserts derived from mouse xCT and 4F2hc cDNAs were produced by PCR reaction using the primers containing mutations to generate appropriate restriction enzyme sites. The sequences of the primers used for PCR were 5′-CTCCTCAGATCTGACACTGCCATGG-3′ and 5′-GTATCTCAATCCTGGGCAGATGGCC-3′ for xCT, and 5′-GCCTCACTGACTACAGATCTTGTCG-3′ and 5′-GGAGGGTGGTAAGCTTTGCATAGGA-3′ for 4F2hc. Both the PCR products were cut out with *Bgl*II and *Hind*III and subcloned into pEGFP-C1 (BD Biosciences Clontech, Palo Alto, CA, USA). The sequences of the final constructs were verified by dideoxy-nucleotide sequencing. Transient transfections were performed using Lipofectamine Plus™ reagents (Invitrogen Corp., Carlsbad, CA, USA) according to the manufacturer's instruction. A2780 cells were plated at 2.5 × 10^5^ in a 35 mm diameter dish and cultured for 24 h. They were transfected for 3 h in Opti-MEM®I (Invitrogen corp., carlsbad, CA, USA), and then the medium was changed to RPMI 1640 with 5% fetal bovine serum and they were incubated for further 33 h.

## RESULTS

### Characterisation of the cystine transport

**Figure 1:**
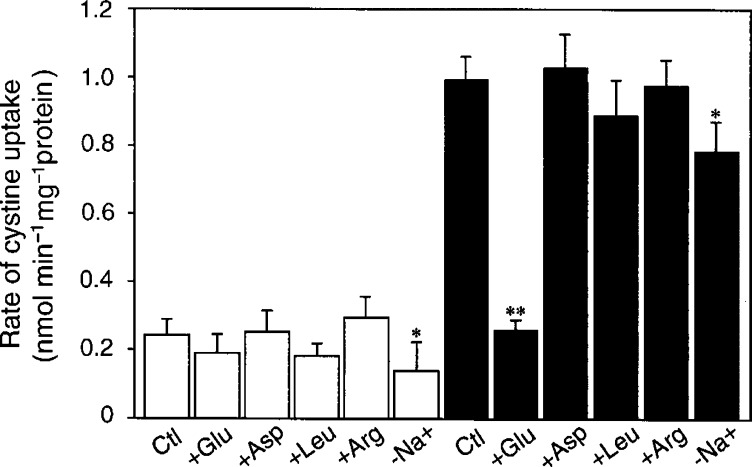
Na^+^-dependency and inhibitory effect of amino acids on the uptake of cystine in A2780 and A2780DDP cells. A2780 (open bars) and A2780DDP cells (filled bars) were cultured in the dish for 24 h and then the rate of cystine (0.05 mM) uptake was measured in the presence of various amino acids (2.5 mM), or in the absence of Na^+^ (−Na^+^). Values are means±s.d. (*n*=4). ^*^Significantly below the corresponding control (Ctl), *P*<0.05; ^**^P<0.01.

As shown in Figure 1

, the rate of cystine uptake was approximately 4.5-fold higher in A2780DDP than that in A2780. To characterise the transport system that mediates the cystine uptake, the rate of cystine uptake was measured in the presence of various amino acids in the uptake medium (Figure 1). In A2780 cells, the uptake of cystine was not inhibited significantly by the amino acids tested. In A2780DDP cells, the uptake of cystine was mostly Na^+^-independent and strongly inhibited by glutamate, but not by aspartate, leucine or arginine. The uptake of cystine in A2780DDP cells appeared to follow saturation kinetics with a *K*_m_ of 0.06 mM whereas no saturation was found in A2780 cells (data not shown). These results show clearly that the resistant cells acquire the cystine transport activity mediated by system x_c_^−^, which does not occur in the sensitive cells.

### Effect of CDDP on the cystine transport

**Figure 2:**
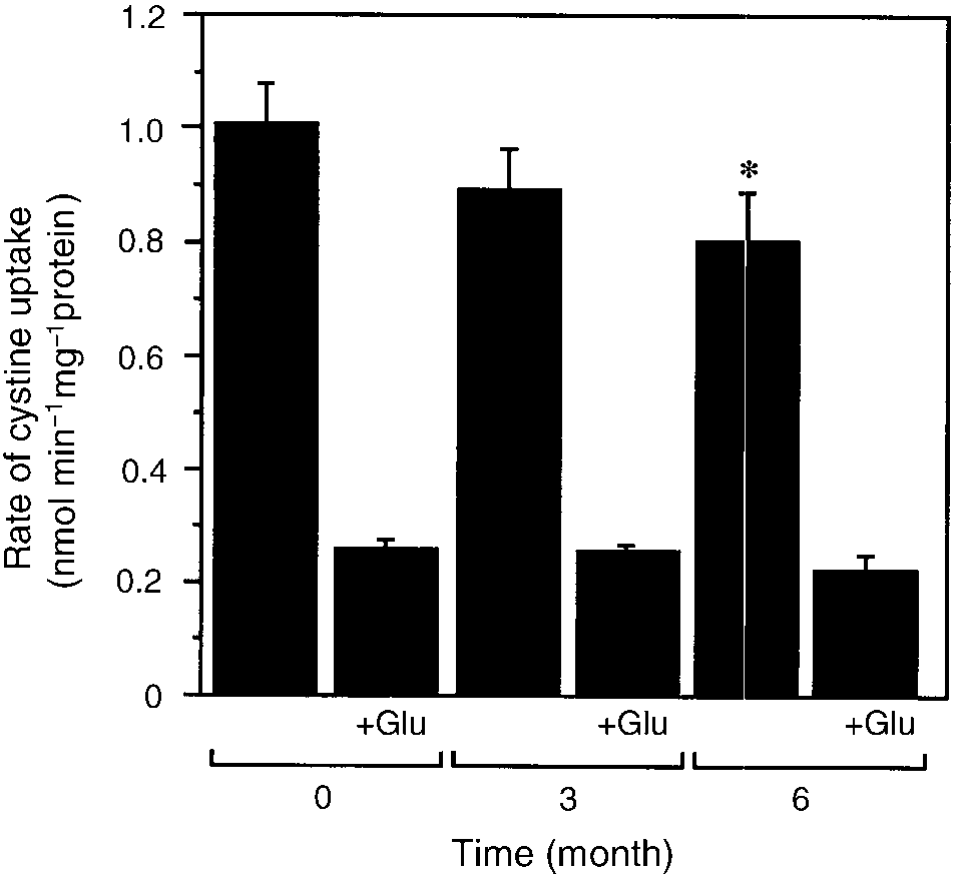
Uptake of cystine in A2780DDP cells cultured without CDDP. A2780DDP cells were cultured without CDDP treatment (see Materials and Methods) for 0, 3 and 6 months. They were plated in the dish and after 24 h the rate of cystine (0.05 mM) uptake was measured. Inhibitory effect of glutamate (2.5 mM) on the cystine uptake was also measured. Values are means±s.d. (*n*=6). ^*^Significantly below control (0 month), *P*<0.05.

In *in vitro* culture, CDDP resistance is not acquired with a single treatment with CDDP but is acquired only after repeated exposure to CDDP for a relatively long time. We examined an effect of CDDP (up to 15 *μ*M) with a single treatment on the cystine uptake in the CDDP-sensitive cells. The cells were incubated for 24 h with various concentrations of CDDP and the cystine uptake was measured. The rate of uptake was not increased by the exposure to CDDP (data not shown). Diethyl maleate (DEM) is an electrophilic agent and is known to induce system x_c_^−^ activity (Bannai, 1984). The rate of cystine uptake in A2780 cells was significantly enhanced (about 1.7-fold increase) by DEM at a single exposure and this enhanced uptake was inhibited by glutamate (data not shown), suggesting that the enhanced uptake is mediated by system x_c_^−^. Then, A2780DDP cells were cultured without CDDP treatment for up to 6 months and the rate of cystine uptake was measured. The rate of cystine uptake decreased slightly but significantly after 6 months without CDDP treatment (Figure 2

). Glutamate was a potent inhibitor of the cystine uptake throughout the experiment, indicating that A2780DDP cells preserve most of the system x_c_^−^ activity for at least 6 months in the absence of CDDP treatment. A2780DDP cells cultured without CDDP treatment for 6 months were tested to see whether they were still resistant to CDDP. When they were incubated with 50 *μ*M CDDP for 24 h, viability was over 95% as judged by dye exclusion, which was nearly equal to that in the control A2780DDP cells. A2780 cells were heavily injured (approximately 5% viable) under such conditions.

### Intracellular GSH levels

**Figure 3:**
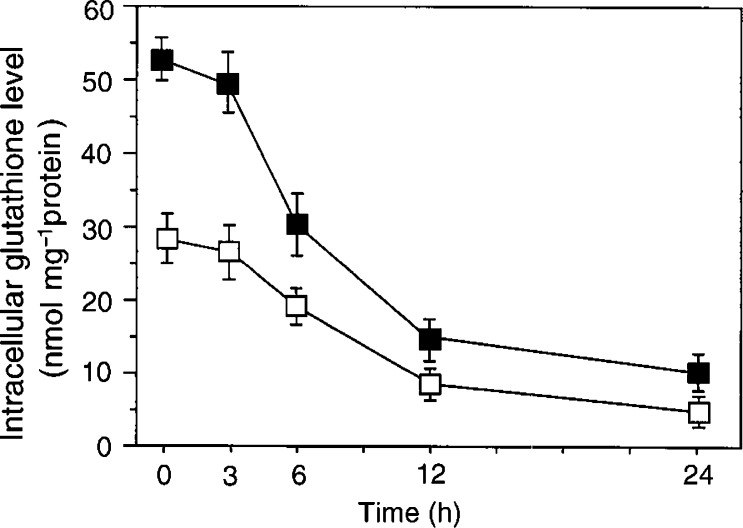
Changes in intracellular GSH levels of A2780 and A2780DDP cells cultured with BSO. A2780 cells (open boxes) and A2780DDP cells (filled boxes) were cultured in the dish for 24 h and then the culture medium was changed to the fresh medium containing 50 *μ*M BSO. They were further cultured for the time indicated and the intracellular GSH levels were determined. Values are means±s.d. (*n*=4).

**Figure 4:**
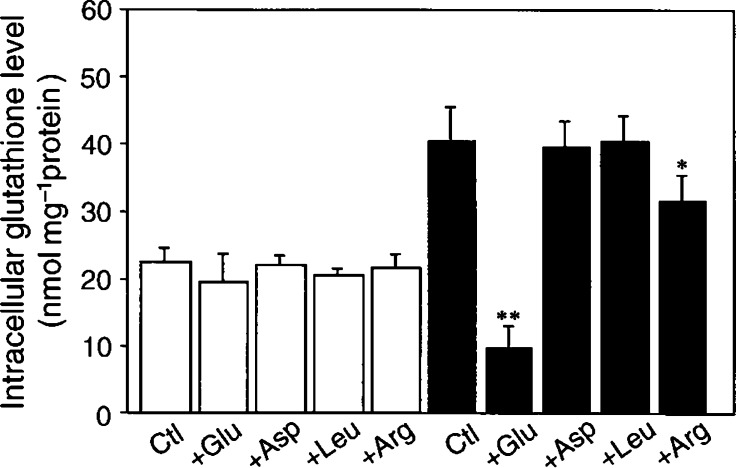
Effect of various amino acids on intracellular GSH levels of A2780 and A2780DDP cells. A2780 cells (open bars) and A2780DDP cells (filled bars) were cultured in the dish for 24 h and then the culture medium was changed to the fresh medium, which was the cystine-restricted (0.05 mM) RPMI1640 with 5% fetal bovine serum and containing the amino acid (10 mM) indicated. The cells were further cultured for 24 h and the intracellular GSH levels were measured. Values are means±s.d. (*n*=4). ^*^Significantly below control (Ctl), *P*<0.05; ^**^P< 0.01.

A2780 and A2780DDP cells were incubated for up to 24 h with 50 *μ*M of buthionine sulphoximine (BSO), which inhibits the GSH synthesis, and the intracellular GSH was measured (Figure 3

). The intracellular GSH level before the incubation with BSO was 1.8-fold higher (*P*<0.01) in A2780DDP (52.5±1.9 nmol per mg protein) than that of A2780 (28.9±2.1 nmol per mg protein). The results are consistent with those reported previously (Goto *et al*, 1995). GSH decreased time dependently and the half-life of intracellular GSH was estimated to be approximately 8–10 h in both cells (Figure 3). Changes in GSH level in the cells incubated for 24 h with excess amino acids were measured (Figure 4

). Since cystine concentration in RPMI 1640 is considerably high (0.2 mM), we used cystine-restricted RPMI 1640 (cystine=0.05 mM) to magnify a possible effect of the amino acids. In A2780DDP cells, the GSH level markedly decreased in the presence of excess glutamate but did not decrease in the presence of excess aspartate or leucine. A slight but significant decrease of an obscure origin was observed in the presence of excess arginine. In A2780 cells, the GSH level did not decrease even in the presence of glutamate. Then, A2780DDP cells were cultured without CDDP treatment for 3 or 6 months, and the intracellular GSH level was measured. The GSH level remained unchanged during six months without CDDP treatment (data not shown).

### Intracellular cysteine levels

**Figure 5:**
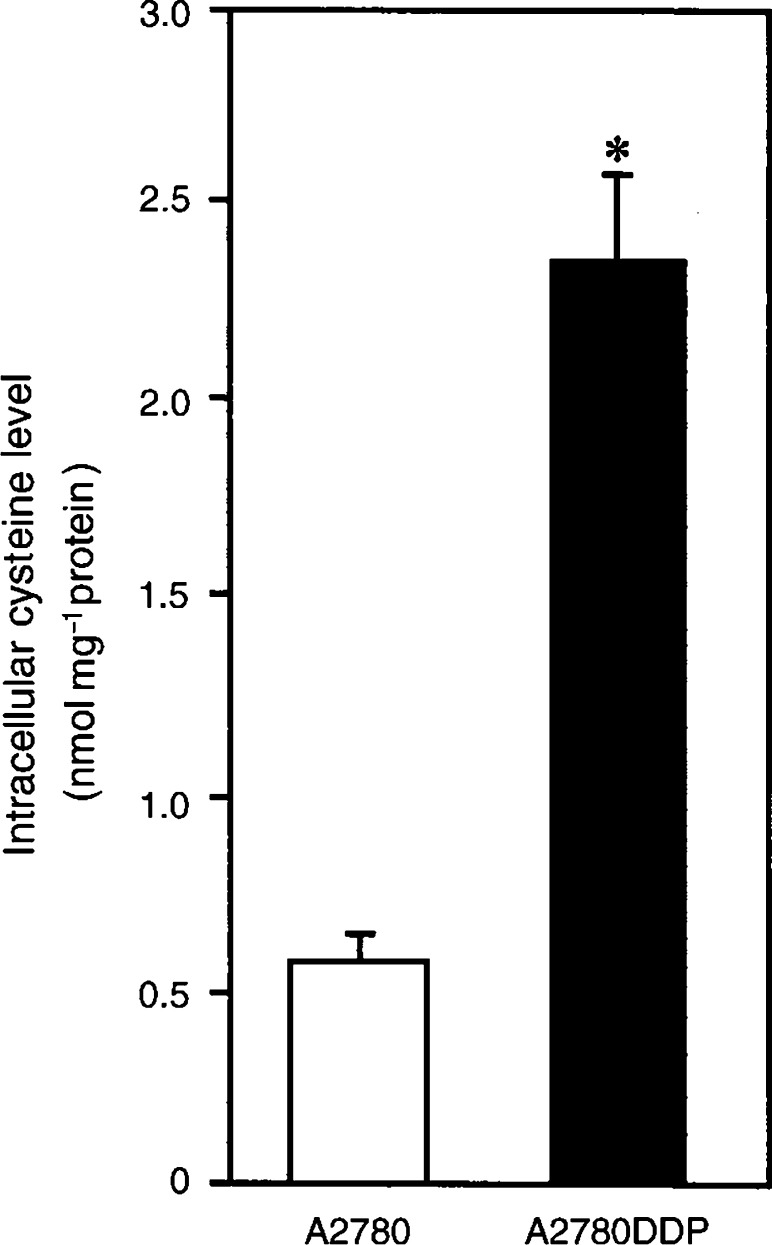
Intracellular cysteine levels in A2780 and A2780DDP cells. A2780 cells (open bars) and A2780DDP cells (filled bars) were cultured in the dish for 24 h and then the culture medium was changed to the fresh one and after 24 h the intracellular cysteine levels were measured. Values are means±s.d. (*n*=4). ^*^Significantly greater than A2780, *P*<0.01.

Intracellular cysteine level in A2780 and A2780DDP is shown in Figure 5

. It was much higher (about 4.2-fold) in the resistant cells than in the sensitive cells. The result suggests that the high content of intracellular GSH in A2780DDP cells is accounted for by the increase in the intracellular content of cysteine, which is a rate-limiting substrate for the GSH synthesis.

### Expression of xCT and 4F2hc mRNAs

**Figure 6:**
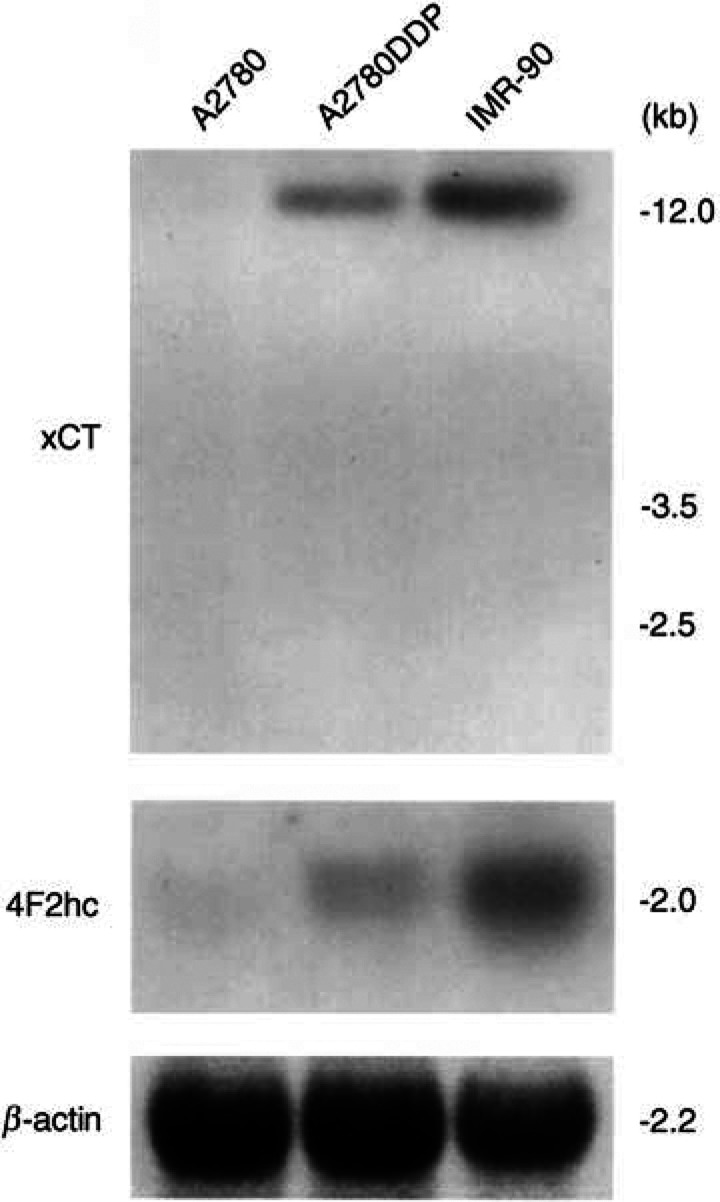
Expression of xCT and 4F2hc mRNAs in A2780 and A2780DDP cells. Twenty micrograms of total RNA isolated from A2780 cells and A2780DDP cells and 10 *μ*g of total RNA isolated from IMR-90 human fibroblasts were loaded. The hybridisation was performed as described in Materials and Methods.

Expression of xCT and 4F2hc mRNAs in A2780 and A2780DDP was examined by Northern blot analysis (Figure 6

). xCT mRNA was hardly detectable in A2780 cells, whereas it was clearly expressed in A2780DDP cells. mRNA for 4F2hc was expressed in both cells, although more strongly in the resistant cells. 4F2hc is not only the component of system x_c_^−^ but also that of some other heterodimeric amino-acid transporters (Palacin *et al*, 1998). IMR-90 human fibroblasts exhibit very high system x_c_^−^ activity and are employed as a positive control. The results clearly showed that system x_c_^−^ was transcriptionally activated in the CDDP-resistant cells.

### CDDP resistance of A2780 cells with xCT expression

**Figure 7:**
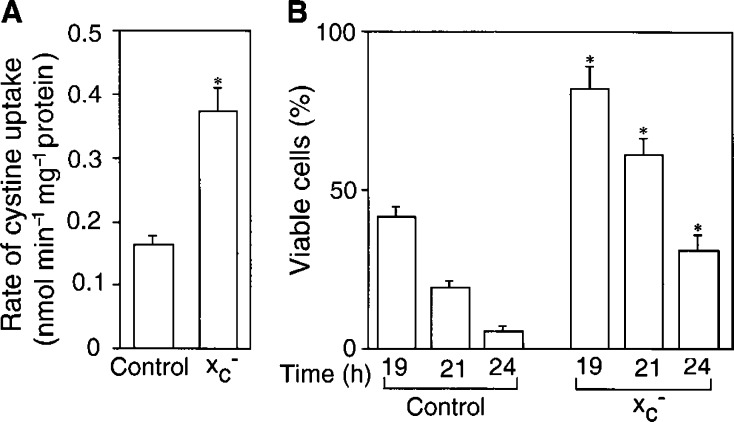
Uptake of cystine and CDDP resistance in A2780 cells transfected with cDNAs for xCT and 4F2hc. A2780 cells were cultured in the dish for 24 h and then they were transfected with pEGFP-C1 (Control) or cotransfected with pEGFP-C1/xCT and pEGFP-C1/4F2hc (x_c_^−^) using Lipofectamine Plus™. After transfection, the cells were incubated for 33 h and the rate of cystine (0.05 mM) uptake was measured (**A**) or CDDP resistance was examined (**B**). CDDP resistance was estimated by measuring viability of the cells treated with 50 *μ*M CDDP in fresh medium for the time indicated. Values are means±s.d. (*n*=6). ^*^Significantly greater than the corresponding control, *P*<0.01.

A2780 cells were transiently transfected with cDNAs of xCT and 4F2hc and the rate of uptake of cystine and CDDP resistance were examined. As shown in Figure 7A

, the rate of uptake in these transfectant cells increased more than twice, and this increased uptake was inhibitable by excess glutamate (data not shown). The cells transfected with the cDNAs were significantly more resistant to CDDP than the control cells (Figure 7B). However, the system x_c_^−^ activity in these transfectants was much lower than that of A2780DDP cells and concomitantly they were less resistant to CDDP than A2780DDP cells. The viability of A2780DDP cells was over 95% when they were incubated in 50 *μ*M CDDP for 24 h.

### Cystine transport activity in human colonic cancer cells

**Figure 8:**
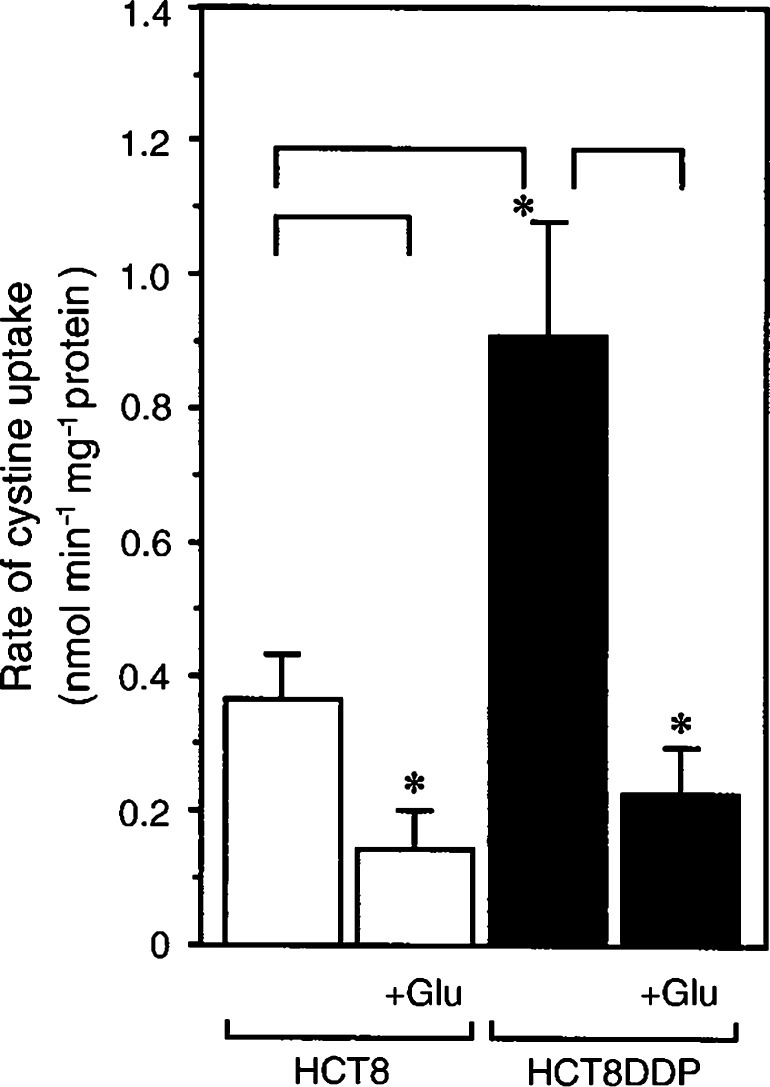
Uptake of cystine in HCT8 and HCT8DDP cells. HCT8 cells (open bars) and HCT8DDP cells (filled bar) were cultured in the dish for 24 h and then the rate of cystine (0.05 mM) uptake was measured in the presence or absence of 2.5 mM of glutamate. Values are means±s.d. (*n*=4). ^*^P<0.01.

As shown in Figure 8

, the rate of cystine uptake was also higher in CDDP-resistant cells derived from human colonic cancer (HCT8DDP cells) than that in the sensitive cells (HCT8 cells). The cystine uptake in HCT8DDP cells was inhibited by glutamate. In the colonic cancer cell lines, unlike the ovarian cancer cells, inhibitory effect of glutamate was observed also in the sensitive cells, indicating the significant occurrence of system x_c_^−^ in HCT8 cells, although its activity is much lower than that of HCT8DDP cells.

## DISCUSSION

Cystine is transported by system x_c_^−^ in the same ionic form as is glutamate, that is, in an anionic form (Bannai and Kitamura, 1981). Homocysteate and *α*-aminoadipate share the transport system but aspartate is not a good substrate. Within physiological pH, cystine occurs as a tetrapolar (neutral as a whole) ion in the main and as a tripolar (anionic as a whole) ion for the rest and the latter is the substrate for system x_c_^−^. System x_c_^−^ is an exchange agency and the physiologic flows via this system are the entry of cystine into the cell coupled with the exit of glutamate from the cell (Bannai, 1986). The cystine uptake through system x_c_^−^ is competitively inhibited by the extracellular glutamate. Another transport system for cystine identified in mammalian plasma membrane is system b^0,+^, which mediates an Na^+^-independent exchange of extracellular cystine and cationic amino acids against intracellular neutral (dipolar) amino acids (Palacin *et al*, 1998). The cystine uptake via this system is competitively inhibited by the extracellular cationic amino acid. Ubiquitous transport systems for neutral amino acids, systems A, ASC and L, usually mediate the transport of dipolar form of the amino acids and cystine is far less reactive with these systems. In the present study, characteristics of the cystine uptake and the expression of xCT and 4F2hc mRNAs in CDDP-resistant cells clearly showed that the cystine uptake in these cells is largely mediated by system x_c_^−^. As shown in Figure 1, the cystine uptake in A2780DDP cells was not inhibited at all by arginine indicating no contribution of system b^0,+^. The CDDP-sensitive A2780 cells also took up cystine, although the rate was much lower than that of the resistant cells (Figure 1). The cystine uptake in A2780 cells was not significantly inhibited by any of the amino acids tested. Thus, neither system x_c_^−^ nor system b^0,+^ mediated the cystine uptake in A2780 cells. At present a transporter, if any, by which cystine is taken up in A2780 cells is unidentified. In HCT8 cells, cystine transport activity was significantly inhibited by glutamate (Figure 8), indicating the occurrence of system x_c_^−^ in these cells, although the resistant variant (HCT8DDP) had a much higher activity of this system.

In the present study, we have found a great increase of cystine transport activity in CDDP-resistant cells (A2780DDP). In these cells, the GSH levels were significantly higher than those in A2780 cells (Figure 3 and Figure 4) and the drastic decrease in GSH by glutamate, a competitive inhibitor of the cystine uptake, indicates that system x_c_^−^ mainly contributes to the high level of GSH in A2780DDP cells. When the GSH synthesis was inhibited by BSO, the half-life of intracellular GSH was nearly equal in both cells (Figure 3). This means that the difference of GSH content in the CDDP-sensitive cells and the CDDP-resistant cells is mainly accounted for by the difference of GSH synthesis rate. The intracellular cysteine level was much higher in A2780DDP cells than in A2780 cells (Figure 8) most probably because of the enhanced uptake of cystine in the former cells. The concentration of cysteine in A2780 cells and A2780DDP cells can be estimated to be approximately 0.11 and 0.46 mM, respectively, assuming that 1 mg cell protein is equivalent to 5 *μ*l cell water. The reported value for *K*_m_ of *γ*-GCS for cysteine is 0.35 mM (Richman and Meister, 1975). Therefore, the rate of GSH synthesis depends almost linearly on the intracellular cysteine level in both A2780 cells and A2780DDP cells. The activity of *γ*-GCS is about two-fold higher in A2780DDP cells than in A2780 cells (Goto *et al*, 1995; Iida *et al*, 1999). It is, thus, reasonably concluded that the high GSH content in CDDP-resistant cells results from the induction of both the system x_c_^−^ transporter and the enzyme *γ*-GCS and that the former contributes more largely to the elevated GSH content than the latter. Despite the considerable increase in both the transport and the enzyme activities, GSH content in A2780DDP cells did not get further than twice in A2780 cells (Figure 3 and Figure 4). This may be accounted for by a feedback inhibition of GSH synthesis by GSH. Goto *et al* (1995) showed that the export of CDDP-GSH adduct was elevated (about 20% increase) in the resistant cells. The value is not large, yet the enhanced export may strengthen the CDDP resistance associated with GSH.

In A2780 cells, the cystine transport activity was not induced by a single exposure to CDDP. In contrast, the cystine transport activity was induced significantly by DEM with the similar treatment. It is obvious that the response to CDDP is quite different from that to DEM. The system x_c_^−^ activity is induced by electrophilic agents like DEM and reaches maximal level at about 48 h (Bannai, 1984). The activity reverts to the original level within 2–3 days if the agent is withdrawn. In contrast, to acquire CDDP resistance the cells are repeatedly treated with CDDP for a long time (Timmer-Bosscha *et al*, 1993). Actually, the sensitive cells are incubated with CDDP for 1 h at a dose inducing approximately 90% cell death and subsequently cultured in fresh medium. After cells are recovered, this treatment is repeated with a stepwise increasing dose of CDDP. It takes about 3–4 weeks for the cell recovery and a long period, about 1 year is required to obtain the CDDP-resistant cell line. Presumably, the cells are selected and acquire the stable and high expression of system x_c_^−^ during this long and repeated exposure to CDDP. In some CDDP-resistant ovarian cancer cell lines, a large number of genetic changes probably associated with the development of CDDP resistance were found (Wasenius *et al*, 1997). The enhanced system x_c_^−^ activity may result from the genetic changes that occurred in CDDP-resistant cells. Recently several genes, which are differentially expressed in association with CDDP resistance, have been identified (Johnsson *et al*, 2000). Among them the genes highly expressed in the resistant cells include cytochrome oxidase I, ribosomal protein 28S, elongation factor 1*α*, *α*-enolase, stathmin and HSP70. The gene for xCT is a novel one and is worthy of note in these studies hereafter.
